# sgRNA amount is a limiting factor in adenine base editing using RNA LNPs

**DOI:** 10.1016/j.omtn.2026.102909

**Published:** 2026-03-18

**Authors:** Alexandra Birkenshaw, Tyler Thomson, Mai P. Truong, Yui Komaki, Nadine Ramsden, Ana Timpano, Cassie Huang, Anna K. Blakney, Daniel Z. Kurek, Jayesh Kulkarni, Colin J.D. Ross

**Affiliations:** 1 Faculty of Pharmaceutical Sciences, University of British Columbia, 2405 Wesbrook Mall, Vancouver, BC V6T 1Z3, Canada; 2 Michael Smith Laboratories, University of British Columbia, Vancouver, BC V6T 1Z4, Canada; 3 School of Biomedical Engineering, University of British Columbia, Vancouver, BC V6T 1Z3, Canada; 4 NanoVation Therapeutics, 2405 Wesbrook Mall, Vancouver, BC V6T 1Z3, Canada; 5 Center for Molecular Medicine and Therapeutics, 950 W 28th Ave, Vancouver, BC V5Z 4H4, Canada; 6 BC Children’s Hospital Research Institute, University of British Columbia, Vancouver, BC, Canada

**Keywords:** MT: RNA/DNA Editing, adenine base editor, ABE, single-guide RNA, sgRNA, on-target editing, tissue specificity, CRISPR/Cas9, gene editing, gene therapy, *in vivo* gene editing, base editing, lipid nanoparticles

## Abstract

Base editors have emerged as powerful tools for precise genome editing, offering significant therapeutic potential. A critical challenge lies in optimizing the delivery and dosage of single-guide RNA to maximize on-target editing efficiency while minimizing off-target and bystander effects. This study investigates the impact of guide RNA dosage on *in vivo* editing efficiency and tissue specificity using a reporter mouse model with a luciferase transgene correctable by adenine base editing. Mice were treated with lipid nanoparticles co-encapsulating a fixed dose of ABE8e RNA and varying doses of guide RNA. Editing outcomes were assessed through whole-body imaging, *ex vivo* tissue analysis, and sequencing. Increasing the guide RNA dose to 4 mg/kg enhanced editing efficiency up to 3.3-fold in multiple tissues compared to the standard 1 mg/kg dose, with the liver exhibiting the highest on-target editing rates at 63%. Bystander editing increased significantly with higher guide RNA doses, particularly in highly edited tissues like the liver, where bystander edits showed a dose-dependent increase. These findings demonstrate the importance of careful guide RNA dose optimization to balance editing efficiency and tissue specificity with bystander effects in the therapeutic applications of ABE8e.

## Graphical abstract

**Figure:**
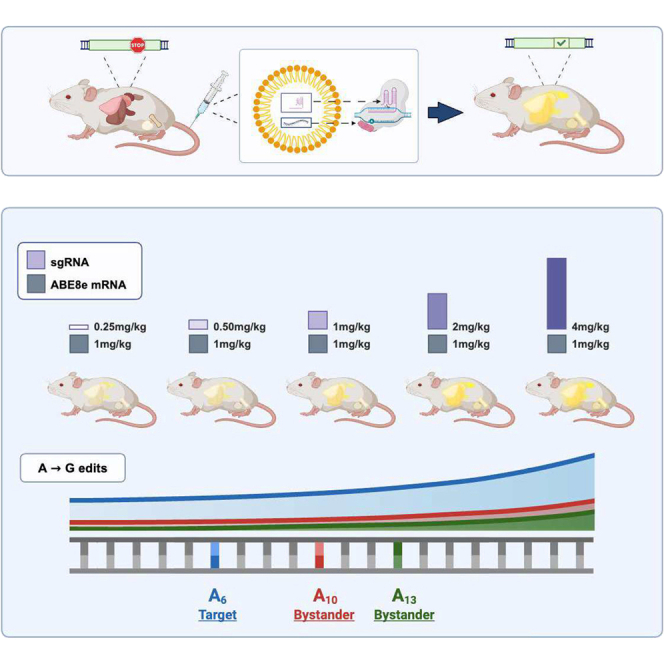

## Teaser

Birkenshaw and colleagues investigate the impact of sgRNA dose on editing rates in two distinct lipid nanoparticle formulations. Increasing sgRNA dose leads to higher on target and bystander editing in most tissues in both formulations representing a simple way to increase editing efficiency.

## Introduction

CRISPR/Cas-derived adenine base editors (ABEs) have transformed the field of genome editing by enabling precise A·T-to-G·C conversions without introducing double-stranded DNA breaks.^1^ This breakthrough offers significant therapeutic potential, as ABEs can theoretically correct approximately one-third of all known disease-causing genetic variants.^2^ These editors are directed to specific genomic sites by single-guide RNAs (sgRNAs), which direct the editing machinery to complementary target sequences.^3^ Despite their potential, the efficient delivery of ABE components remains a critical challenge.

In recent years, lipid nanoparticles (LNPs) have emerged as an effective strategy for delivering mRNA and sgRNA. LNPs facilitate cellular internalization and protect nucleic acids from nuclease degradation after systemic injection.^4^ Clinical trials utilizing LNPs to deliver ABEs are currently underway (NCT05398029).^5^ Upon cellular entry, ABE mRNA is translated into protein via ubiquitous cellular machinery. Successful base editing requires the translated ABE and sgRNA to form a bimolecular complex in the cytosol before translocating to the nucleus.^6^ However, after systemic injection, sgRNA is cleared from tissues and plasma to undetectable levels within 72 h.^7^ This instability presents a unique challenge, as the concentration of nuclear sgRNA directly limits editing efficiency.^8^ To address this, strategies such as chemical modifications like 2′-O-methyl and phosphorothioate bonds, which are commonly employed to protect sgRNA from exonuclease degradation, and circularization of sgRNA have been employed, improving stability and enhancing editing outcomes.^9^^,^^10^

Despite these advances, the optimal ratio of ABE mRNA to sgRNA for *in vivo* editing remains poorly understood. While most studies use a 1:1 mass ratio (Table 1), a limited body of evidence suggests that the ideal ratio may depend on factors such as sgRNA stability and tissue type. For example, one study found that minimally modified sgRNAs achieve peak editing efficiency at a 3:1 mRNA:sgRNA ratio, whereas extensively modified sgRNAs perform best at a 1:1 ratio. The authors found that the extensively modified sgRNA had a longer half-life and hypothesized that lower doses of sgRNA are needed for more robust sgRNA.^11^ A different study using AAVs observed tissue-specific effects of sgRNA dose, with reduced sgRNA levels decreasing editing efficiency in the heart and muscle but not the liver.^28^ These inconsistencies highlight the need for a deeper understanding of how mRNA:sgRNA ratios influence editing outcomes.

**Table 1:** mRNA-to-sgRNA ratios used for *in vivo* gene editing delivered by LNPs

Title	Editor type	Gene target	mRNA:sgRNA mass ratio	Ratio optimization explored?
A single administration of CRISPR/Cas9 lipid nanoparticles achieves robust and persistent *in vivo* genome editing^7^	CRISPR Cas9	TTR	1:1	no
High-throughput *in vivo* screen of functional mRNA delivery identifies nanoparticles for endothelial cell gene editing^11^	CRISPR Cas9	ICAM2	1:1	tested 1:1, 3:1, and 5:1. In both spleen and liver cells, more indel formation at a 1:1 ratio rather than 5:1.
*In vivo* CRISPR base editing of PCSK9 durably lowers cholesterol in primates^12^	ABE8.8	PCSK9	1:1	no
Ionizable lipid nanoparticles for therapeutic base editing of congenital brain disease^13^	ABE7.10	IDUA	1:1	yes. *In vitro* optimization using 3:1, 1:1, 1:3. no significant difference across the 3 groups.
*In vivo* cytidine base editing of hepatocytes without detectable off-target mutations in RNA and DNA^14^	saKKH-CBE3 mRNA	PAH	1:1	no
Optimization of LNP for *in vivo* base editing^15^	ABE	–	1:1	no
Evaluation of cytosine base editing and adenine base editing as a potential treatment for alpha-1 antitrypsin deficiency^16^	ngcABEvar9 mRNA CBE4 mRNA	*SERPINA1*	1:1	no
A base editing strategy using mRNA-LNPs for *in vivo* correction of the most frequent phenylketonuria variant^17^	ABE8.8	PAH	1:1	no
Rapid and definitive treatment of phenylketonuria in variant-humanized mice with corrective editing^18^	ABE8.8	PAH	1:1	no
LNP-mediated delivery of CRISPR RNP for widespread *in vivo* genome editing in mouse cornea^19^	CRISPR Cas9	PAX6	1:1	no
Guanidinium-rich lipopeptide-based nanoparticle enables efficient gene editing in skeletal muscles^20^	CRISPR Cas9	DMD	1:1	no
Preformed vesicle approach to LNP manufacturing enhances retinal mRNA delivery^21^	CRISPR Cas9	*LoxP*	1:1	no
*In vivo* delivery of CRISPR-Cas9 using lipid nanoparticles enables antithrombin gene editing for sustainable hemophilia A and B therapy^22^	CRISPR Cas9	Serpinc1	1:1	no
Lipid-nanoparticle-mediated codelivery of Cas9 mRNA and single-guide RNA achieves liver-specific *in viv*o genome editing of Angptl3^23^	CRISPR Cas9	*Angptl3*	1:1.2	yes. Tested 2:1, 1:1.2 and 1:2 with no significant difference, proceeded with 1:1.2.
Adenine base editing in an adult mouse model of tyrosinemia^24^	ABERA6.3	FAH	2:1	no
Minimizing the ratio of ionizable lipid in lipid nanoparticles for *in vivo* base editing^25^	ABE8.8 m	PCSK9	2:1	no
Structure-guided chemical modification of guide RNA enables potent non-viral *in vivo* genome editing^26^	CRISPR Cas9	GFP, PCSK9, FAH, ROSA26	2.4:1	no
Membrane destabilizing ionizable phospholipids for organ-selective mRNA delivery and CRISPR/Cas gene editing^27^	CRISPR Cas9	PTEN	4:1	no

To address this knowledge gap, we investigated the impact of varying ABE8e mRNA:sgRNA ratios using two LNP formulations with distinct lipid compositions: (1) a formulation with lipid ratios similar to the clinically approved Onpattro (ONP), which predominantly targets the liver, and (2) a proprietary LNP formulation (NVTxLNP) commercialized by NanoVation Therapeutics containing adjusted lipid ratios to facilitate broader tissue expression.^29^^,^^30^

NVTxLNP utilizes an increased phospholipid content to encourage longer circulation time and extrahepatic delivery. Increased phospholipid content results in particles with a unique protein corona and lower adsorption of surface proteins compared to Onpattro-like formulations.^30^ The resulting nanoparticles circulate for longer with reduced accumulation in the liver and spleen, allowing them to reach a broader range of tissues.^29^^,^^30^ By comparing these formulations, we aimed to determine whether the effect of mRNA:sgRNA ratios depends on LNP composition or tissue type.

A fixed ABE8e mRNA dose of 1 mg/kg was administered, while the sgRNA doses ranged from 0.25 to 4 mg/kg, ensuring that any observed changes in editing efficiency were attributable to the variations in the amount of sgRNA rather than the ABE8e.

To evaluate editing efficiency, we utilized a luminescent ABE reporter mouse model (LumA), which contains an ABE-correctable nonsense mutation in a luciferase transgene.^31^ Correction of this mutation restores functional luciferase production, enabling whole-body quantification of gene editing through luminescence imaging. The system has a high degree of sensitivity, with editing rates of under 1% resulting in detectable luminescent signal; however, the degradation of proteins can vary greatly between tissues, which limits the ability to quantitatively compare editing rates in different tissues solely based on luminescence. To address this limitation, all tissues that showed luminescence were sequenced with next-generation sequencing (NGS). This approach provides a robust platform to assess the influence of mRNA:sgRNA ratios on base editing efficiency across tissues and LNP formulations, providing insights for optimizing therapeutic genome editing.

## Results

### Delivery and biodistribution

**Figure 1:**
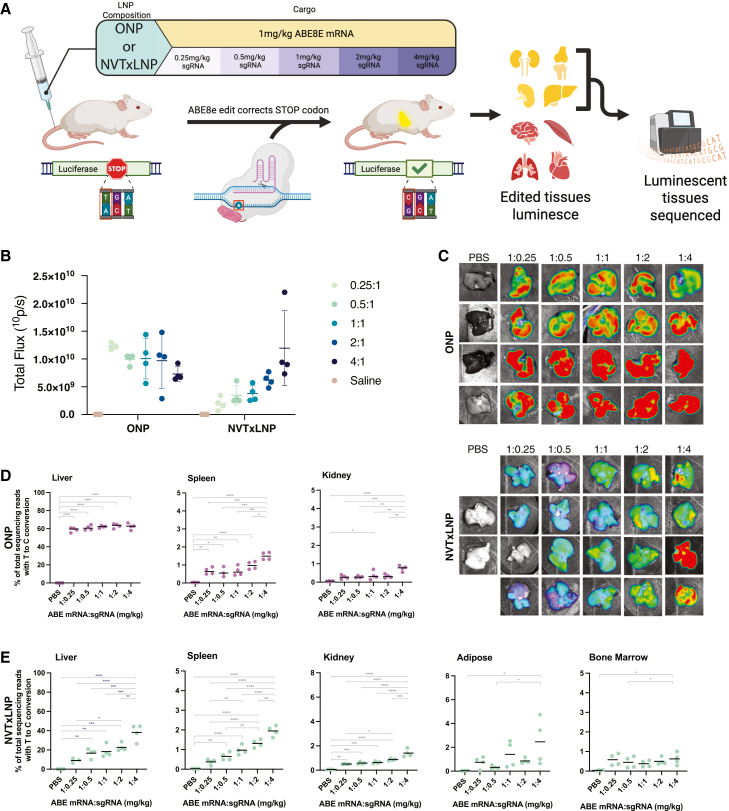
Impact of sgRNA dose on *in vivo* editing (A) Schematic of the experimental design. Reporter mice carried a luciferase transgene containing a premature stop codon. Successful correction by ABE8e restored luminescence in edited tissues. Two types of lipid nanoparticles (LNPs) were used to co-encapsulate ABE8e mRNA and sgRNA. ABE8e mRNA was fixed at 1 mg/kg, while sgRNA was dosed at 0.25, 0.5, 1, 2, or 4 mg/kg. (B) Quantification of whole-body luminescence 14 days post-treatment. Mean (SD) (*n* = 4). (C) *Ex vivo* imaging of liver tissues 14 days after treatment. (D) Mean (SD) editing efficiency in luminescent tissues from ONP-treated mice. Mean (SD) (*n* = 4 for all groups, except spleen of 1:0.5, which is *n* = 3). (E) Mean (SD) editing efficiency in luminescent tissues from NVTxLNP-treated mice. Significance was determined by ordinary one-way ANOVA with multiple-comparisons test (∗*p* ≤ 0.05, ∗∗*p* ≤ 0.01, ∗∗∗*p* ≤ 0.001, ∗∗∗∗*p* ≤ 0.0001).

Two LNP formulations were employed to deliver ABE8e mRNA and sgRNA payloads intravenously to the luminescent reporter mice (Figure 1A). The size, polydispersity index, and encapsulation efficiency of LNPs were unaffected by the ratio of sgRNA:mRNA (Figure S1A). Although the final encapsulated mass ratio was not directly quantified, the input ratios are inferred to be maintained post-formulation based on two key observations. First, high encapsulation efficiencies were consistently achieved across all formulations, suggesting minimal loss of cargo during the formulation process. Second, comparative analysis of single-cargo LNPs (containing only mRNA or sgRNA) versus co-encapsulated formulations demonstrated equivalent encapsulation rates, suggesting that the relative encapsulation of the two species remains equivalent when co-encapsulated (Figure S1B). Modifications of sgRNA were limited to the terminal three nucleotides at either end of the sgRNA for this study, as we did not observe any benefit to using highly modified sgRNA with internal modifications (Figure S2).

Luminescent signals in the LumA model plateau within 2 weeks following injections; as such, editing was assessed 14 days after treatment.^31^ Whole-body live imaging showed a positive correlation between luminescent signal intensity and sgRNA dose in the NVTxLNP-treated mice, indicating an increase in editing, but no change was observed in the ONP group (Figure 1B). Tissue-specific *ex vivo* imaging (Figure 1C) and luciferase assays (Figures S3 and S4) showed editing in a variety of tissues. The liver, spleen, and kidney of ONP-treated mice and the liver, spleen, kidney, adipose, and bone marrow of NVTxLNP-treated mice had luminescent signals above background levels in the luciferase assay (Figures S3 and S4) and were further analyzed using NGS to precisely quantify editing efficiency (Figures 1D and 1E).

### Dose-dependent increase in on-target editing across extrahepatic tissues

The ONP formulation demonstrated canonical LNP tropism, with editing primarily observed in the liver, spleen, and kidney. In contrast, the NVTxLNP formulation exhibited broader biodistribution, with additional editing detected in adipose tissue and bone marrow (Figure 1E). Using a one-way ANOVA with a multiple comparisons test for linear trends, we found that increasing the amount of sgRNA resulted in higher on-target editing in most tissues, except in the liver of ONP-treated mice (Figures 1D and 1E; Table S1).

Compared to the standard 1:1 ABE mRNA:sgRNA ratio, a 1:4 ratio significantly increased editing efficiency in multiple tissues. In the spleen, editing increased 2.25-fold (*p* = 0.0151) for ONP-treated mice and 2.01-fold (*p* = 0.00352) for NVTxLNP-treated mice (Figures 1D and 1E). Similarly, editing in the kidney increased 2.08-fold (*p* = 0.0721) for ONP-treated mice and 3.27-fold for NVTxLNP-treated mice (*p* = 0.00129). In NVTxLNP-treated mice, editing in adipose tissue and bone marrow increased 1.74- and 1.65-fold, respectively, but was not significant according to a Welch *t* test due to a high degree of variability. Interestingly, ONP-treated livers showed no significant changes in editing efficiency across the tested ratios, while NVTxLNP-treated livers exhibited a 2.08-fold (*p* = 0.0105) increase at a 1:4 ratio (Figures 1D and 1E).

### Off-target editing analysis

To assess off-target editing, the top two guide-dependent off-target sites predicted by CRISTA^32^ and COSMID^33^ were examined. These programs use machine learning and computer algorithms to predict sequence sites where unintended off-target editing is likely to occur. We sequenced these sites in the liver of ONP-treated mice, which exhibited the highest on-target editing rates. No off-target editing was detected at the two sites examined (Figure S5).

### Bystander editing is increased at higher sgRNA doses

**Figure 2:**
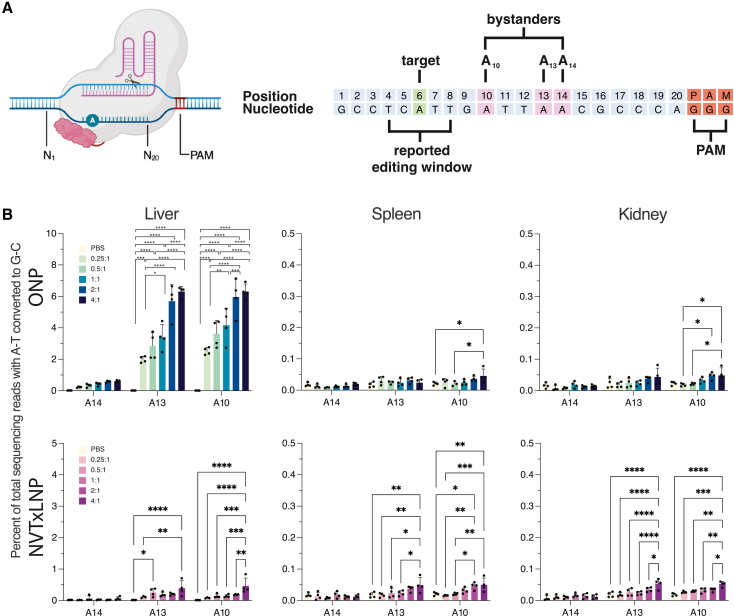
Assessment of bystander editing (A) Nucleotides are numbered starting 21 bases upstream of the PAM site. The target base is highlighted in green, while potential bystander bases are shown in purple. (B) Editing rates of bystander editing at positions A10, A13, and A14. Mean (SD) significance was determined by two-way ANOVA with Bonferroni’s multiple comparison test (∗*p* ≤ 0.05, ∗∗*p* ≤ 0.01, ∗∗∗*p* ≤ 0.001, ∗∗∗∗*p* ≤ 0.0001).

Bystander editing, which refers to the unintended editing of adenines proximate to the target base, was evaluated at three positions (A10, A11, and A14) near the target adenine (A6) within the protospacer (Figure 2A). ABE8e's canonical editing window is considered to be A4–A8.^1^

In NVTxLNP-treated mice, bystander editing was detected at positions A10 and A13 in the liver, spleen, and kidney but not in adipose tissue or bone marrow. Bystander editing rates were modest (<0.5%), with higher sgRNA doses resulting in greater bystander editing. This is seen in the NVTxLNP-treated livers where editing at A10 was 0.464% ± 0.252% for mice treated with a 1:4 ratio, representing a 3.2-fold (*p* = 0.05) increase compared to the 1:1 group (Figure 2B). ONP-treated mice exhibited higher bystander editing rates, with values reaching up to 6% in the liver (Figure 2B). Bystander edits were also observed at A10 in the spleen and kidney of ONP-treated mice, though no editing was detected at A13 or A14 in these tissues.

Interestingly, the bystander editing was approximately 5% of the on-target editing across all tissues and treatments, with the exception of the liver in ONP-treated mice, where increasing sgRNA doses led to a higher proportion of bystander edits (Figure S6).

### Indel formation

The livers of both ONP-treated and NVTxLNP-treated mice showed significant rates of indel formation compared to the PBS group (Figure S8). No other tissues had detectable levels of indels. The livers of the ONP-treated mice in the highest sgRNA dose group had the highest levels of indels at 1.48% (±0.038%). An ordinary one-way ANOVA with multiple comparisons testing for trends showed a dose-dependent increase in indel formation in the livers of ONP mice (*p* = 0.002) but the trend in NVTxLNP mice was not significant (*p* = 0.2735).

## Discussion

These findings demonstrate the significant impact of modulation of the sgRNA dose for increasing both on-target and bystander editing. Increasing sgRNA doses significantly enhanced on-target editing across multiple tissues; however, in ONP-treated livers, editing efficiency plateaued at approximately 60%, with no further improvement observed at higher sgRNA doses (Figure 1D). This saturation likely reflects the limit of transfectable hepatocytes via the ApoE-mediated endocytosis pathway utilized by ONP-like LNPs. Once the maximum fraction of accessible cells are edited, increasing the cargo load provides no additional benefit for on-target editing. Similar editing ceilings have been reported in other studies using LNP-delivered base editors.^15^^,^^28^^,^^34^ Another study using AAV-delivered base editors found that increasing the sgRNA dose increased editing in tissues such as the heart and skeletal muscle but not in the liver, which had already reached 60% editing.^28^

Previous studies have delivered a fixed total dose of RNA while varying the ratio of editor mRNA:sgRNA. This makes it difficult to elucidate whether changes in editing can be attributed to a decrease in sgRNA or an increase in editor mRNA. We fixed the ABE8e mRNA dose at 1 mg/kg while varying the sgRNA dose from 0.25 to 4 mg/kg. However, it is important to note that this leads to a difference in total RNA dose and lipid load in different treatment groups. There were no adverse effects seen in animal behavior and condition after treatment, with the exception of weight. All animals in the ONP group had weight loss of under 5%. However, two animals in the highest dose group of the NVTxLNP had weight loss of between 5% and 10% but recovered by 4 days post-injection (Figure S7). This weight loss is likely attributable to the acute immune response triggered by the high total lipid and RNA load.

While we did not observe detectable off-target edits at the top two predicted sites, we acknowledge that limiting analysis to the top two predicted sites is a constraint of this study as prediction models are not always accurate. Future studies on sgRNA dose would benefit from un-biased, genome-wide off-target screening methods.

Bystander editing was about 5% of on-target editing in most tissues (Figure S6). In ONP-treated livers, where we suspect on-target editing had reached its maximum, higher sgRNA doses resulted in increased bystander editing as the editor began targeting adjacent adenines. This suggests that the editor becomes more promiscuous when the canonical editing window becomes fully edited. A previous study examined the editing window of NG-ABE8e and found high editing rates of up to A12 when no other editable bases were present in the editing window.^34^

The originally described editing window of ABE8e is A4–A8^1^; however, other studies have reported broader windows of A3–A11.^35^ Notably, we found that ABE8e′s editing window extends beyond the previously documented A4–A8 range, with detectable edits reaching A14 (Figure 2). This broader range increases the risk of unintended bystander mutations, making it crucial to account for potential bystander edits up to A14 when designing sgRNA for ABE8e applications. An ideal sgRNA design places the intended edit at an optimal location within the editing window while considering the impact of bystander editing of other bases within the editing window. At some targets, bystanders can introduce missense or nonsense mutations, which would reverse any benefit of on-target editing. Current algorithms often prioritize the placement of the target adenine within A4–A8. Our data suggest that for ABE8e, design algorithms should also screen for and penalize potential bystander adenines up to position A14 to ensure safety.

While higher sgRNA doses improved editing efficiency, they also increased the risk of bystander editing, particularly in tissues where editing efficiency neared its maximum. A similar trend was observed for indel formation. Indel rates were detectable exclusively in the livers of both LNPs, with only the ONP-treated livers showing a statistically significant dose-dependent increase in indel formation (Figure S8). This emphasizes the need for dose optimization to balance editing efficiency with specificity as once on-target editing is saturated, high doses could result in unintended bystander edits and indel formation.

Overall, our results also highlight sgRNA as a limiting factor when using LNPs to co-deliver ABE8e and sgRNA. The mRNA must first be translated into protein before it can form functional RNP complexes, during which time sgRNA can be degraded. Strategies to enhance sgRNA stability and resistance to exonuclease degradation could improve its functional availability without requiring higher doses. Given the cost-effectiveness of sgRNA production compared to mRNA, optimizing its dosage can enhance both the efficacy and accessibility of CRISPR-based therapies.

We suspect that our findings, especially the dose-dependent increase in editing, would apply to other base editors with spCas9 domains since sgRNA complexes with the Cas domain rather than interacting with the deaminase. High-fidelity, narrow-windowed ABE variants such as ABE8.8 or ABE7.10 may be less likely to see the expanded editing window we observed in this study. Further studies to assess potential differences in the effect of different editors or alternative Cas domains would be useful.

While our results demonstrate that increasing sgRNA dose can be an effective way to boost on-target editing, our safety assessment was limited to body-condition and weight monitoring. Future work examining serum biomarkers for hepatotoxicity (ALT/AST) and histological analysis (H&E) would be required to comprehensively define the safety profile of high-dose sgRNA administration prior to therapeutic administration.

This study provides critical insights into the relationship between sgRNA dosage, editing efficiency, and bystander effects, offering a framework for optimizing base editor systems for therapeutic applications. By addressing the challenges of sgRNA stability and dosage, future work can further refine the precision and safety of genome editing technologies.

## Materials and methods

### ABE mRNA and gRNA

ABE8e mRNA with m1Ψ modifications was synthesized by NanoVation Therapeutics. The sequence is as described by Dr. David Liu’s group. The sgRNA was purchased from Integrated DNA Technologies. The following sequence was used: mG∗mC∗mC∗ rUrCrA rUrUrG rArUrU rArArC rGrCrC rCrArGrUrUrU rUrArG rArGrC rUrArG rArArA rUrArG rCrArA rGrUrU rArArA rArUrA rArGrG rCrUrA rGrUrC rCrGrU rUrArU rCrArA rCrUrU rGrArA rArArA rGrUrG rGrCrA rCrCrG rArGrU rCrGrG rUrGrC mU∗mU∗mU∗ rU, where r_ is an unmodified RNA base, m_ is a 2′O-methyl modified RNA base, and m_∗ is a phosphorothioated 2′-O-methyl RNA base.

### LNP formulation and characterization

LNPs were prepared by injecting aqueous and organic phases through a Tee-mixer at a 3:1 volume ratio (aqueous:organic) with a 15 and 5 mL/min flow rate, respectively. Lipids were dissolved in ethanol, and mRNA/sgRNA was mixed in 25 mM sodium acetate (pH 4) at specified ratios. Formulations were dialyzed in Spectra/Por 2 dialysis tubing with a 12–14 kD MWCO with 1,000-fold 1× PBS (pH 7.4) for 16 h at room temperature. They were concentrated by centrifugation at 1,500 g in Amicon Ultra-15 centrifugal filter units. Size and PDI were measured using a Malvern Zetasizer Nano at 4.65 nm. Encapsulation was measured using a RiboGreen assay. Both formulations use a proprietary ionizable lipid developed by NanoVation Therapeutics.

### Animal experiments

Mouse experiments were performed adhering to protocols approved by the UBC Animal Care Committee. Four lumA mice were used per treatment group, two males and two females. All mice were between 8 and 12 weeks old. Tail vein injections at a volume of 10 mL/kg were given at the following doses for each group: 1.25 mg/kg (1 mg/kg ABE8e mRNA, 0.25 mg/kg sgRNA), 1.5 mg/kg (1 mg/kg ABE8e mRNA, 0.5 mg/kg sgRNA), 2 mg/kg (1 mg/kg ABE8e mRNA, 1 mg/kg sgRNA), 3 mg/kg (1 mg/kg ABE8e mRNA, 2 mg/kg sgRNA), and 5 mg/kg (1 mg/kg ABE8e mRNA, 4 mg/kg sgRNA).

Animals were live imaged 48 h post-injection at 7 and 14 dpi. Animals were injected with D-luciferin (Cayman Chemicals, CAY14681) dissolved in 1× DPBS at a total dose of 150 mg/kg, 15 min before imaging with a LagoX imager. On day 14, mice were sacrificed via deep isoflurane anesthesia followed by cervical dislocation. Livers, lungs, spleens, kidneys, hearts, adipose, brains, and muscles were collected, imaged, and snap-frozen in liquid nitrogen. Bone marrow was harvested and put on ice for immediate processing via luciferase assay.

### Tissue processing

A Steady-Glo Luciferase assay (Promega E2520) was performed on all collected tissues. Tissues were weighed, and an amount of GLO Lysis buffer was added based on the amount of tissue to ensure a constant dilution factor within each tissue while adding a sufficient volume to ensure the tubes did not break or overflow: liver: 1 mL/g; adipose, brain, heart, kidney, and muscle: 2 mL/g; lung: 3 mL/g; and spleen: 10 mL/g. Tissues were homogenized with a FastPrep homogenizer (MP Biomedicals) at speed 6 for three rounds of 20 s. Homogenate was diluted 6× in Glo lysis buffer. Equal parts of homogenate and Steady Glo Lysis buffer were added to a white plate, and luminescence was measured on a plate reader. Note: tissues from ONP-treated mice and NVTxLNP-treated mice were measured on different plate readers with different sensitivities due to a flood that destroyed the initial machine.

All tissues with Luciferase Assay signals above the background were sequenced. DNA was extracted from tissues using a DNeasy Blood & Tissues kit (QIAGEN 69506) according to the manufacturer’s protocols. A sequencing library was prepared by a two-step PCR using the following primers with NGS sequencing adaptors to amplify the section of interest (TCG TCG GCA GCG TCA GAT GTG TAT AAG AGA CAG ACA CCC GAG GGG GAT GAT AA and GTC TCG TGG GCT CGG AGA TGT GTA TAA GAG ACA GCG CCA TCC TTG TCA ATC AAG GCG), then adding index barcodes in a second PCR. Sequencing was done to a minimum of 10,000× coverage using an Illumina MiSeq with a read length of 2 × 150 bp and analyzed with CRISPResso2.^14^

### Statistical analysis

Statistical analysis was performed in GraphPad Prism.

## Data and code availability

All data that support the conclusions of this article can be found in the main text or the supplemental material. LumA mice are available under materials transfer agreements (MTAs) with the University of British Columbia (UBC) under the stock number JAX Stock No. 038165 LumA.

## Acknowledgments

The authors thank members of the Ross Lab, Tessa Morin, Liam O’Keeffe, and Danil Timofeyev, for their helpful discussions and suggestions in the preparation of this article. Research in this publication was supported by 10.13039/501100000233Genome British Columbia project code SIP005, 10.13039/501100000245Michael Smith Foundation for Health Research Scholar Award #16458, and 10.13039/100020797Nanomedicines Innovation Network (NMIN) grant 2019-T2-05. C.J.D.R. is a 10.13039/501100000245Michael Smith Foundation for Health Research Scholar. A.B. is a MITACs Accelerate fellowship at NanoVation Therapeutics. Images were created with BioRender.

## Author contributions

C.J.D.R., A.B., and T.T. conceived the research. A.B., T.T., Y.K., and N.R. performed the experiments. D.Z.K. and J.K. provided LNP formulations. This work was supervised by C.J.D.R., D.Z.K., J.K., and A.K.B. A.B., M.P.T., T.T., and A.T. wrote the original draft, and C.J.D.R., A.B., M.P.T., T.T., N.R., C.H., D.Z.K., and A.K.B. reviewed and edited the manuscript.

## Declaration of interests

D.Z.K. and J.K. have financial interests in NanoVation Therapeutics. A.B. is a MITACs Accelerate Intern at NanoVation Therapeutics. The other authors have no competing interests.

## Supplemental information

Document S1. Figures S1–S8 and Tables S1 and S2

Document S2. Article plus supplemental information

## References

[1] Richter M.F., Zhao K.T., Eton E., Lapinaite A., Newby G.A., Thuronyi B.W., Wilson C., Koblan L.W., Zeng J., Bauer D.E. (2020). Phage-assisted evolution of an adenine base editor with improved Cas domain compatibility and activity. Nat. Biotechnol..

[2] Landrum M.J., Lee J.M., Benson M., Brown G., Chao C., Chitipiralla S., Gu B., Hart J., Hoffman D., Hoover J. (2016). ClinVar: public archive of interpretations of clinically relevant variants. Nucleic Acids Res..

[3] Gaudelli N.M., Komor A.C., Rees H.A., Packer M.S., Badran A.H., Bryson D.I., Liu D.R. (2017). Programmable base editing of A T to G C in genomic DNA without DNA cleavage. Nature.

[4] Hou X., Zaks T., Langer R., Dong Y. (2021). Lipid nanoparticles for mRNA delivery. Nat. Rev. Mater..

[5] Horie T., Ono K. (2024). VERVE-101: a promising CRISPR-based gene editing therapy that reduces LDL-C and PCSK9 levels in HeFH patients. Eur. Heart J. Cardiovasc. Pharmacother..

[6] Allen D., Rosenberg M., Hendel A. (2020). Using Synthetically Engineered Guide RNAs to Enhance CRISPR Genome Editing Systems in Mammalian Cells. Front. Genome Ed.

[7] Finn J.D., Smith A.R., Patel M.C., Shaw L., Youniss M.R., Van Heteren J., Dirstine T., Ciullo C., Lescarbeau R., Seitzer J. (2018). A Single Administration of CRISPR/Cas9 Lipid Nanoparticles Achieves Robust and Persistent In Vivo Genome Editing. Cell Rep..

[8] Ma H., Tu L.-C., Naseri A., Huisman M., Zhang S., Grunwald D., Pederson T. (2016). CRISPR-Cas9 nuclear dynamics and target recognition in living cells. J. Cell Biol..

[9] Javaid N., Choi S. (2021). CRISPR/Cas System and Factors Affecting Its Precision and Efficiency. Front. Cell Dev. Biol..

[10] Barber H.M., Pater A.A., Gagnon K.T., Damha M.J., O’Reilly D. (2025). Chemical engineering of CRISPR–Cas systems for therapeutic application. Nat. Rev. Drug Discov..

[11] Sago C.D., Lokugamage M.P., Paunovska K., Vanover D.A., Monaco C.M., Shah N.N., Gamboa Castro M., Anderson S.E., Rudoltz T.G., Lando G.N. (2018). High-throughput in vivo screen of functional mRNA delivery identifies nanoparticles for endothelial cell gene editing. Proc. Natl. Acad. Sci. USA.

[12] Musunuru K., Chadwick A.C., Mizoguchi T., Garcia S.P., DeNizio J.E., Reiss C.W., Wang K., Iyer S., Dutta C., Clendaniel V. (2021). In vivo CRISPR base editing of PCSK9 durably lowers cholesterol in primates. Nature.

[13] Palanki R., Bose S.K., Dave A., White B.M., Berkowitz C., Luks V., Yaqoob F., Han E., Swingle K.L., Menon P. (2023). Ionizable Lipid Nanoparticles for Therapeutic Base Editing of Congenital Brain Disease. ACS Nano.

[14] Villiger L., Rothgangl T., Witzigmann D., Oka R., Lin P.J.C., Qi W., Janjuha S., Berk C., Ringnalda F., Beattie M.B. (2021). In vivo cytidine base editing of hepatocytes without detectable off-target mutations in RNA and DNA. Nat. Biomed. Eng..

[15] Chen D. (2021).

[16] Packer M.S., Chowdhary V., Lung G., Cheng L.-I., Aratyn-Schaus Y., Leboeuf D., Smith S., Shah A., Chen D., Zieger M. (2022). Evaluation of cytosine base editing and adenine base editing as a potential treatment for alpha-1 antitrypsin deficiency. Mol. Ther..

[17] Brooks D.L., Whittaker M.N., Said H., Dwivedi G., Qu P., Musunuru K., Ahrens-Nicklas R.C., Alameh M.-G., Wang X. (2024). A base editing strategy using mRNA-LNPs for in vivo correction of the most frequent phenylketonuria variant. HGG Adv..

[18] Brooks D.L., Carrasco M.J., Qu P., Peranteau W.H., Ahrens-Nicklas R.C., Musunuru K., Alameh M.-G., Wang X. (2023). Rapid and definitive treatment of phenylketonuria in variant-humanized mice with corrective editing. Nat. Commun..

[19] Mirjalili Mohanna S.Z., Djaksigulova D., Hill A.M., Wagner P.K., Simpson E.M., Leavitt B.R. (2022). LNP-mediated delivery of CRISPR RNP for wide-spread in vivo genome editing in mouse cornea. J. Contr. Release.

[20] Zhu M., Wang X., Xie R., Wang Y., Xu X., Burger J., Gong S. (2023). Guanidinium-Rich Lipopeptide-Based Nanoparticle Enables Efficient Gene Editing in Skeletal Muscles. ACS Appl. Mater. Interfaces.

[21] Eygeris Y., Henderson M.I., Curtis A.G., Jozić A., Stoddard J., Reynaga R., Chirco K.R., Su G.L.-N., Neuringer M., Lauer A.K. (2024). Preformed Vesicle Approach to LNP Manufacturing Enhances Retinal mRNA Delivery. Small.

[22] Han J.P., Kim M., Choi B.S., Lee J.H., Lee G.S., Jeong M., Lee Y., Kim E.-A., Oh H.-K., Go N. (2022). In vivo delivery of CRISPR-Cas9 using lipid nanoparticles enables antithrombin gene editing for sustainable hemophilia A and B therapy. Sci. Adv..

[23] Qiu M., Glass Z., Chen J., Haas M., Jin X., Zhao X., Rui X., Ye Z., Li Y., Zhang F., Xu Q. (2021). Lipid nanoparticle-mediated codelivery of Cas9 mRNA and single-guide RNA achieves liver-specific in vivo genome editing of *Angptl3*. Proc. Natl. Acad. Sci. USA.

[24] Song C.-Q., Jiang T., Richter M., Rhym L.H., Koblan L.W., Zafra M.P., Schatoff E.M., Doman J.L., Cao Y., Dow L.E. (2020). Adenine base editing in an adult mouse model of tyrosinaemia. Nat. Biomed. Eng..

[25] Chen Q., Wang X., Zhang Y., Tian M., Duan J., Zhang Y., Yin H. (2024). Minimizing the ratio of ionizable lipid in lipid nanoparticles for in vivo base editing. Natl. Sci. Rev..

[26] Yin H., Song C.-Q., Suresh S., Wu Q., Walsh S., Rhym L.H., Mintzer E., Bolukbasi M.F., Zhu L.J., Kauffman K. (2017). Structure-guided chemical modification of guide RNA enables potent non-viral in vivo genome editing. Nat. Biotechnol..

[27] Liu S., Cheng Q., Wei T., Yu X., Johnson L.T., Farbiak L., Siegwart D.J. (2021). Membrane-destabilizing ionizable phospholipids for organ-selective mRNA delivery and CRISPR-Cas gene editing. Nat. Mater..

[28] Davis J.R., Wang X., Witte I.P., Huang T.P., Levy J.M., Raguram A., Banskota S., Seidah N.G., Musunuru K., Liu D.R. (2022). Efficient in vivo base editing via single adeno-associated viruses with size-optimized genomes encoding compact adenine base editors. Nat. Biomed. Eng..

[29] Chander N., Basha G., Yan Cheng M.H., Witzigmann D., Cullis P.R. (2023). Lipid nanoparticle mRNA systems containing high levels of sphingomyelin engender higher protein expression in hepatic and extra-hepatic tissues. Mol. Ther., Methods Clin. Dev..

[30] Cheng M.H.Y., Zhang Y., Fox K., Leung J., Strong C., Kang E., Chen Y., Tong M., Bommadevara H., Jan E. (2025). Liposomal lipid nanoparticles for extrahepatic delivery of mRNA. Nat. Commun..

[31] Yu S.-Y., Carlaw T., Thomson T., Birkenshaw A., Basha G., Kurek D., Huang C., Kulkarni J., Zhang L.-H., Ross C.J.D. (2023). A luciferase reporter mouse model to optimize in vivo gene editing validated by lipid nanoparticle delivery of adenine base editors. Mol. Ther..

[32] Abadi S., Yan W.X., Amar D., Mayrose I. (2017). A machine learning approach for predicting CRISPR-Cas9 cleavage efficiencies and patterns underlying its mechanism of action. PLoS Comput. Biol..

[33] Cradick T.J., Qiu P., Lee C.M., Fine E.J., Bao G. (2014). COSMID: A Web-based Tool for Identifying and Validating CRISPR/Cas Off-target Sites. Mol. Ther. Nucleic Acids.

[34] Tu T., Song Z., Liu X., Wang S., He X., Xi H., Wang J., Yan T., Chen H., Zhang Z. (2022). A precise and efficient adenine base editor. Mol. Ther..

[35] Lee S.-H., Wu J., Im D., Hwang G., Jeong Y.K., Jiang H., Lee S.J., Jo D.H., Goddard W.A., Kim J.H. (2024). Bystander base editing interferes with visual function restoration in Leber congenital amaurosis. Preprint at Genetics.

