# Supplementary material for: sgRNA amount is a limiting factor in adenine base editing using RNA LNPs
Source: Mol Ther Nucleic Acids. 2026 Mar 18;37(2):102909. doi: 10.1016/j.omtn.2026.102909 (PMC13090319; doi:10.1016/j.omtn.2026.102909)
Supplement: Document S1. Figures S1–S8 and Tables S1 and S2 [file mmc1.pdf]

OMTN, Volume *37*

Supplemental information

sgRNA amount is a limiting factor

in adenine base editing using RNA LNPs

Alexandra Birkenshaw, Tyler Thomson, Mai P. Truong, Yui Komaki, Nadine Ramsden, Ana Timpano, Cassie Huang, Anna K. Blakney, Daniel Z. Kurek, Jayesh Kulkarni, and Colin J.D. Ross

Supplemental Information

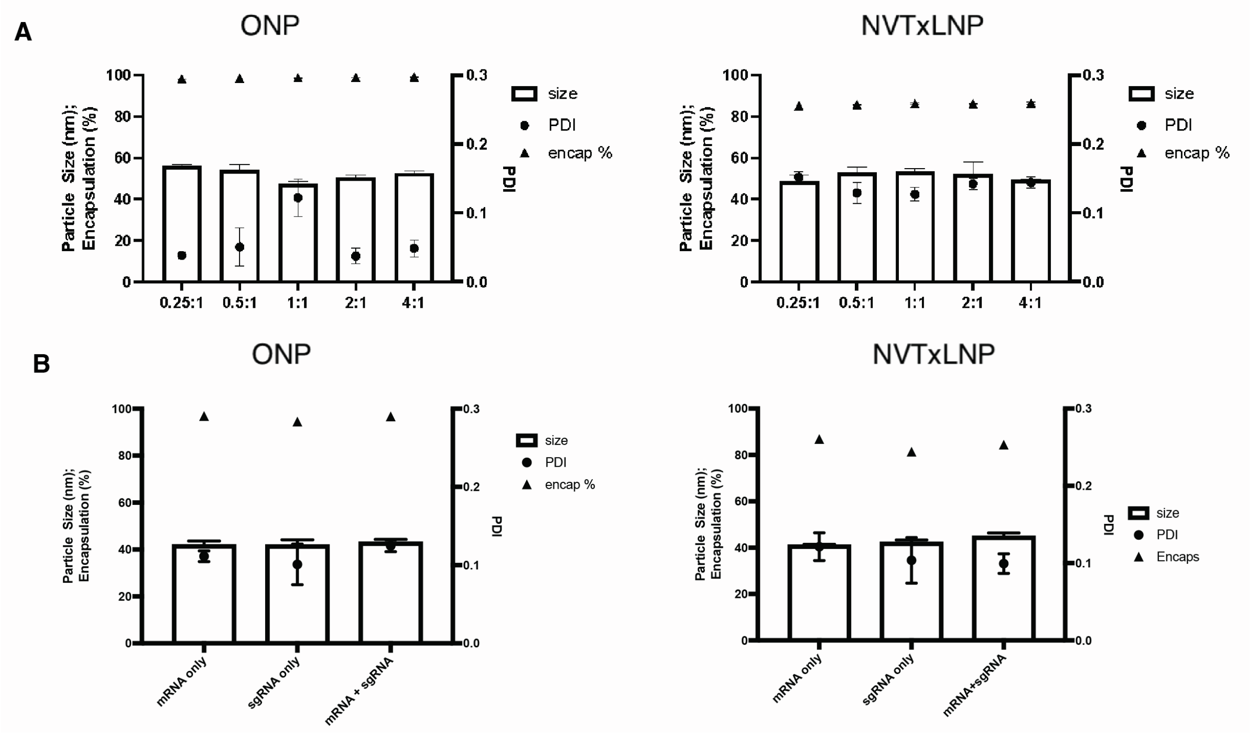

Figure S1. Physicochemical characteristics of lipid nanoparticles. Size and PDI were measured using a Malvern Zetasizer Nano at 4.65nm. Encapsulation was measured using a ribogreen assay. Data represents mean (SD), N=2. **A)** Characteristics of LNPs for *in vivo* injection **B)** Characteristics of LNPs to compare encapsulation

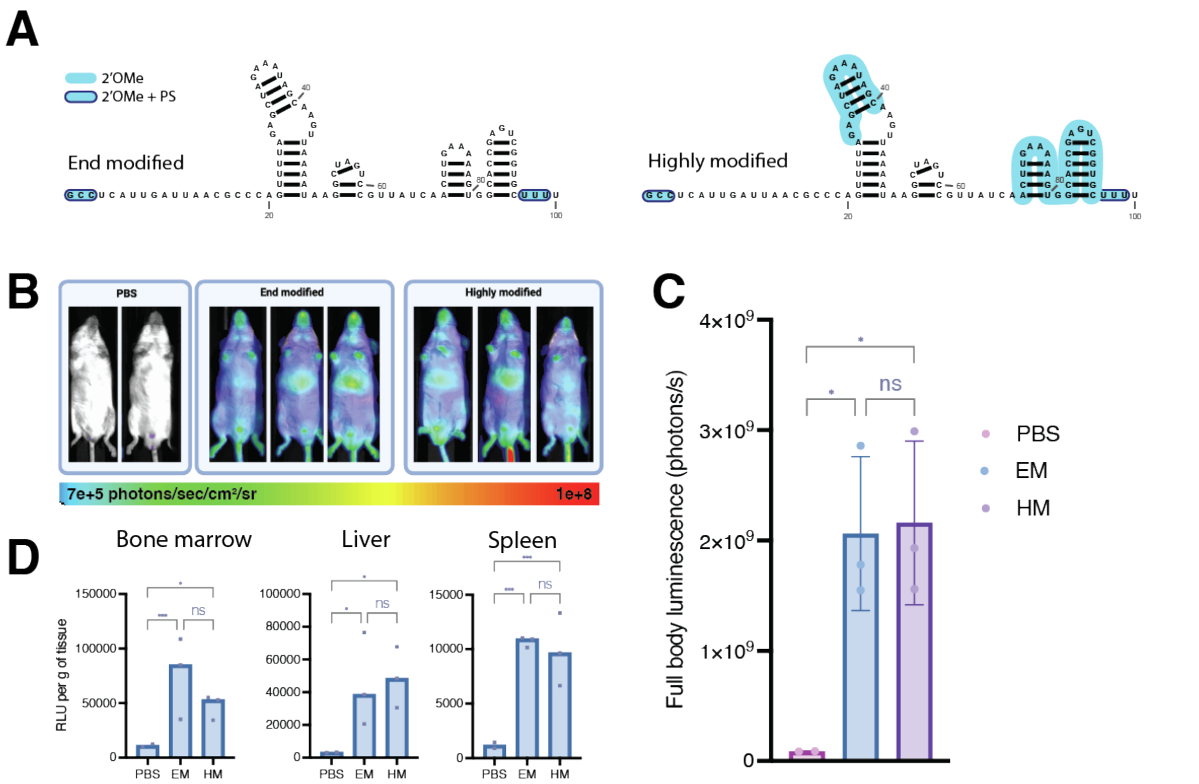

Figure S2. Comparison of end-modified (EM) vs highly-modified (HM) sgRNA. A) Schematic showing the modifications of each sgRNA. **B)** Full body IVIS images of lumA mice injected at 1mg/kg of LNP containing sgRNA and ABE8e mRNA at a 1:1 ratio **C)** Full body luminescence quantified by IVIS imaging **D)** Luciferase assay results from homogenized tissues. Data represents the mean. Statistics determined by two-way ANOVA with Tukey's multiple comparisons test.

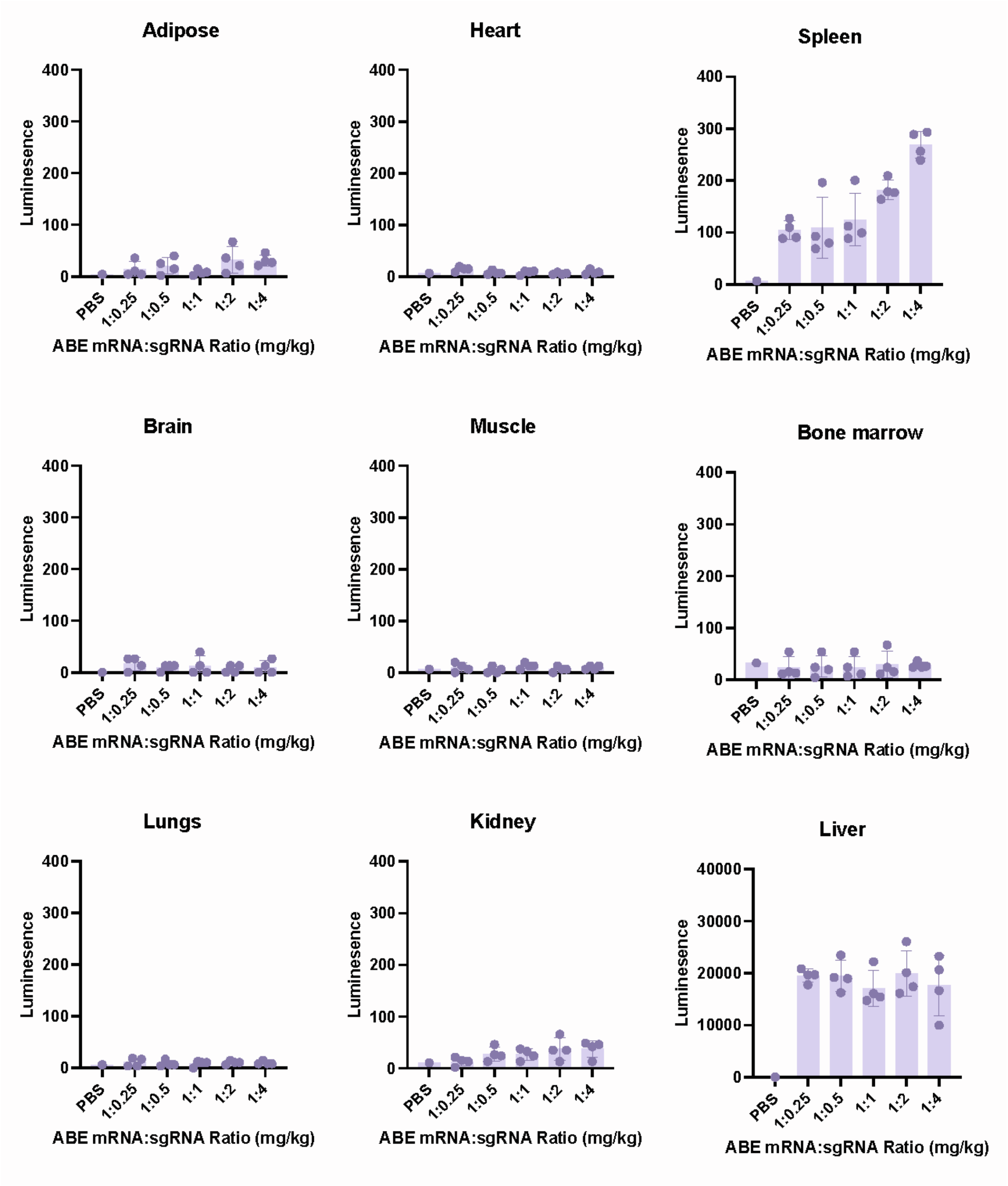

Figure S3. Luciferase assay of ONP-treated mice. Data represents the mean (SD), N=4.

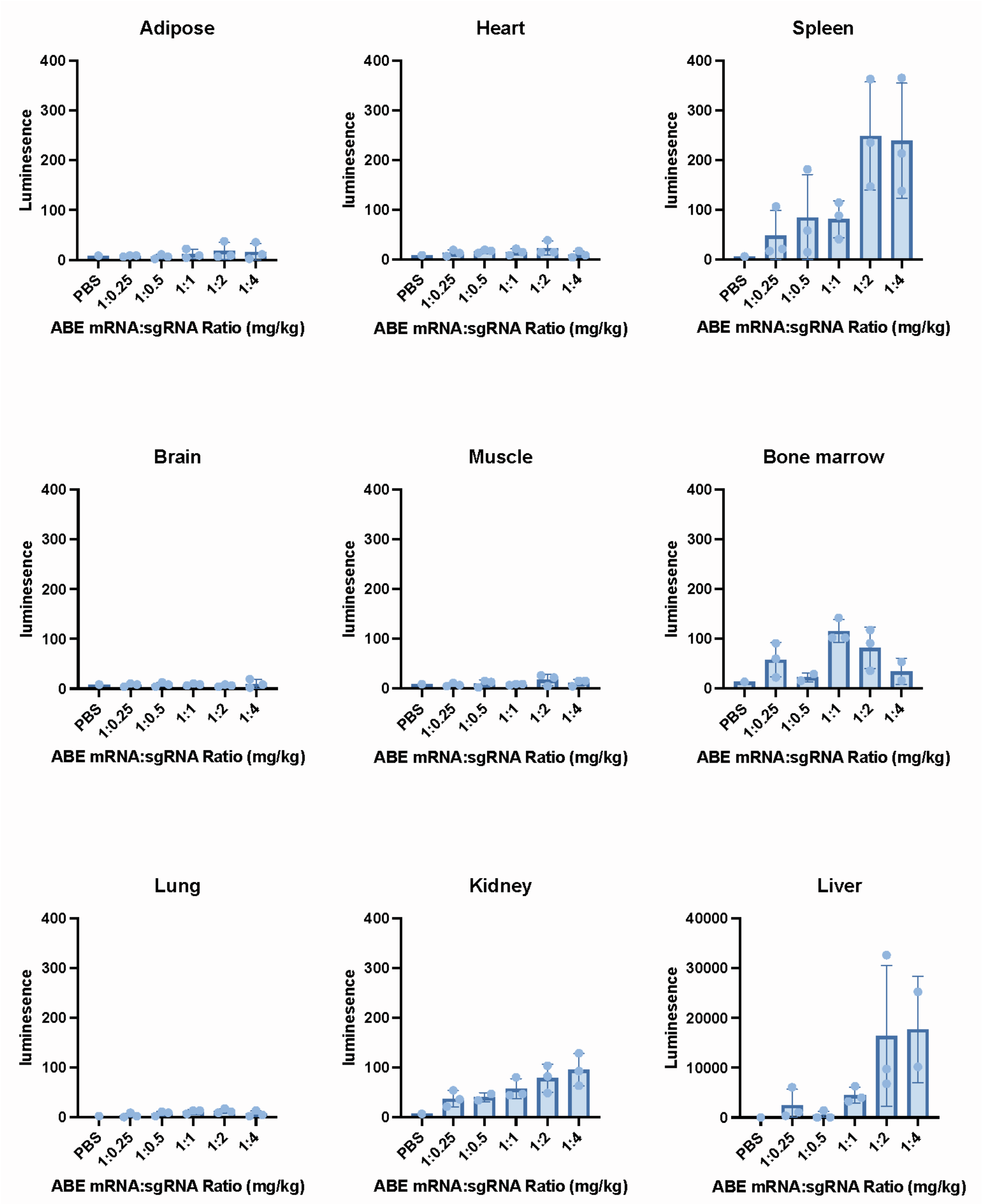

Figure S4. Luciferase Assay from NVTxLNP-treated mice. Data represents the mean (SD), N=4.

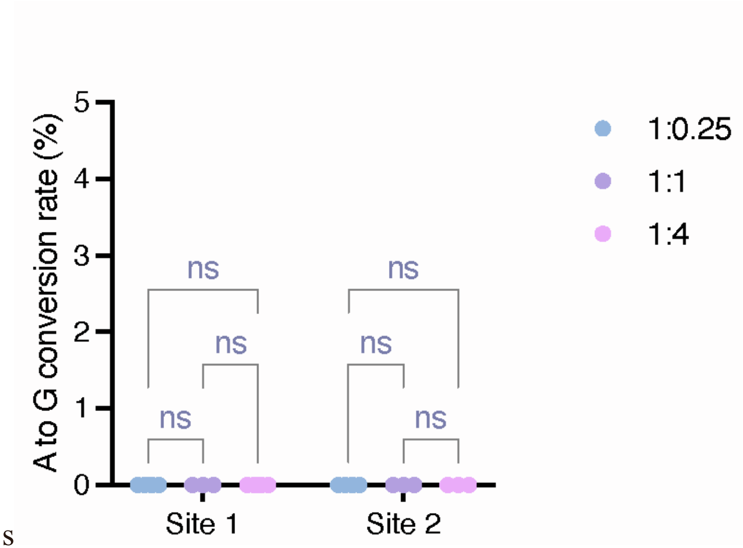

s

Figure S5. Off-target editing in the liver at the top two predicted off-target sites. Data represents mean (SD). Significance was determined by two-way ANOVA with Bonferroni's multiple comparisons test.

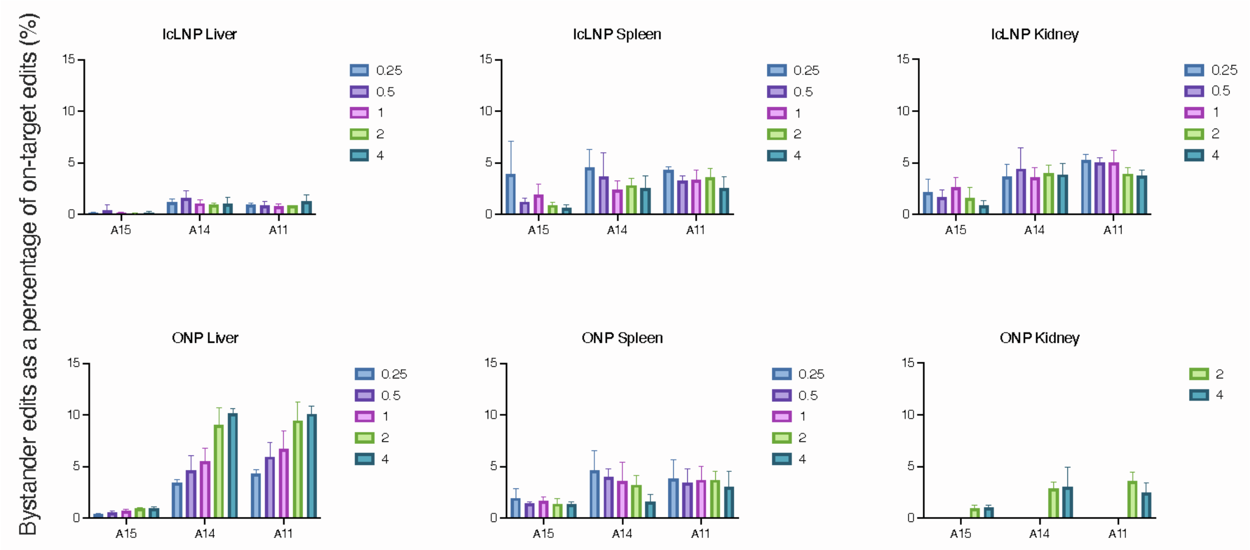

Figure S6. Bystander edits vs. on-target edits. Bystander editing is shown as a percentage of on-target editing rates for the same treatment group. Only treatments with significant on-target editing rates, as determined by a two-way ANOVA with Bonferroni's multiple comparisons test and 95% CI, are shown. Data represents mean (SD) with n=4.

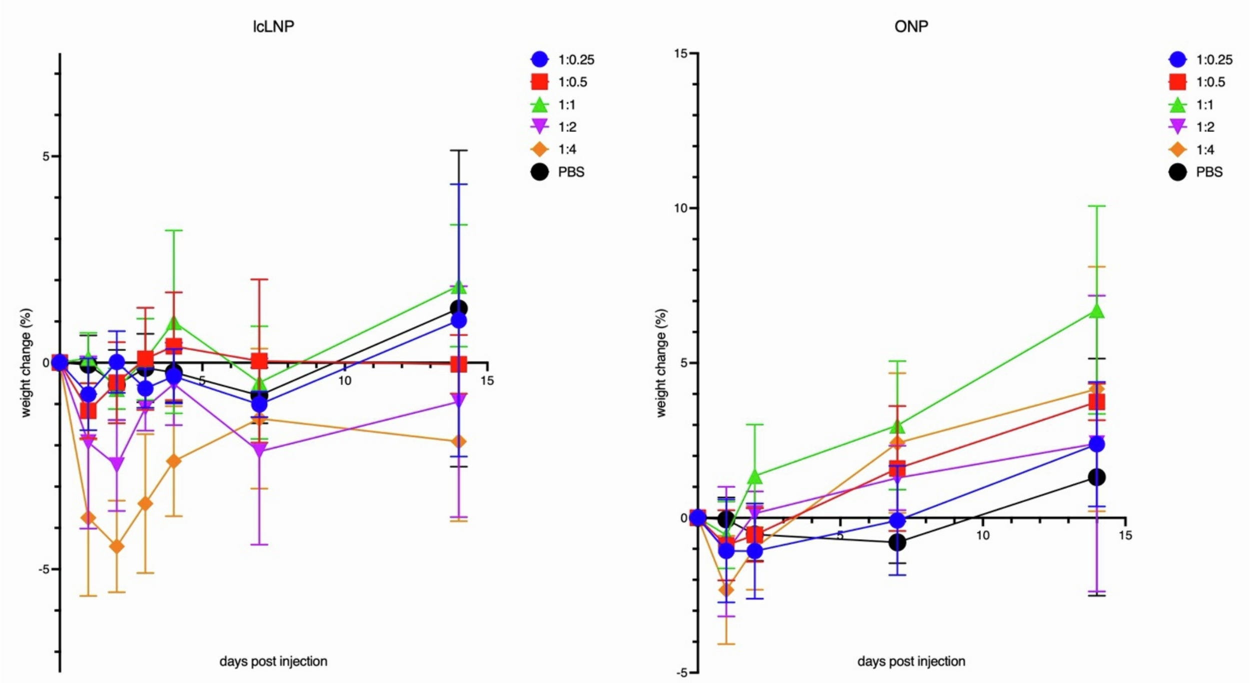

Figure S7: Percentage weight change after treatment: Data represents the mean with SD (N=4).

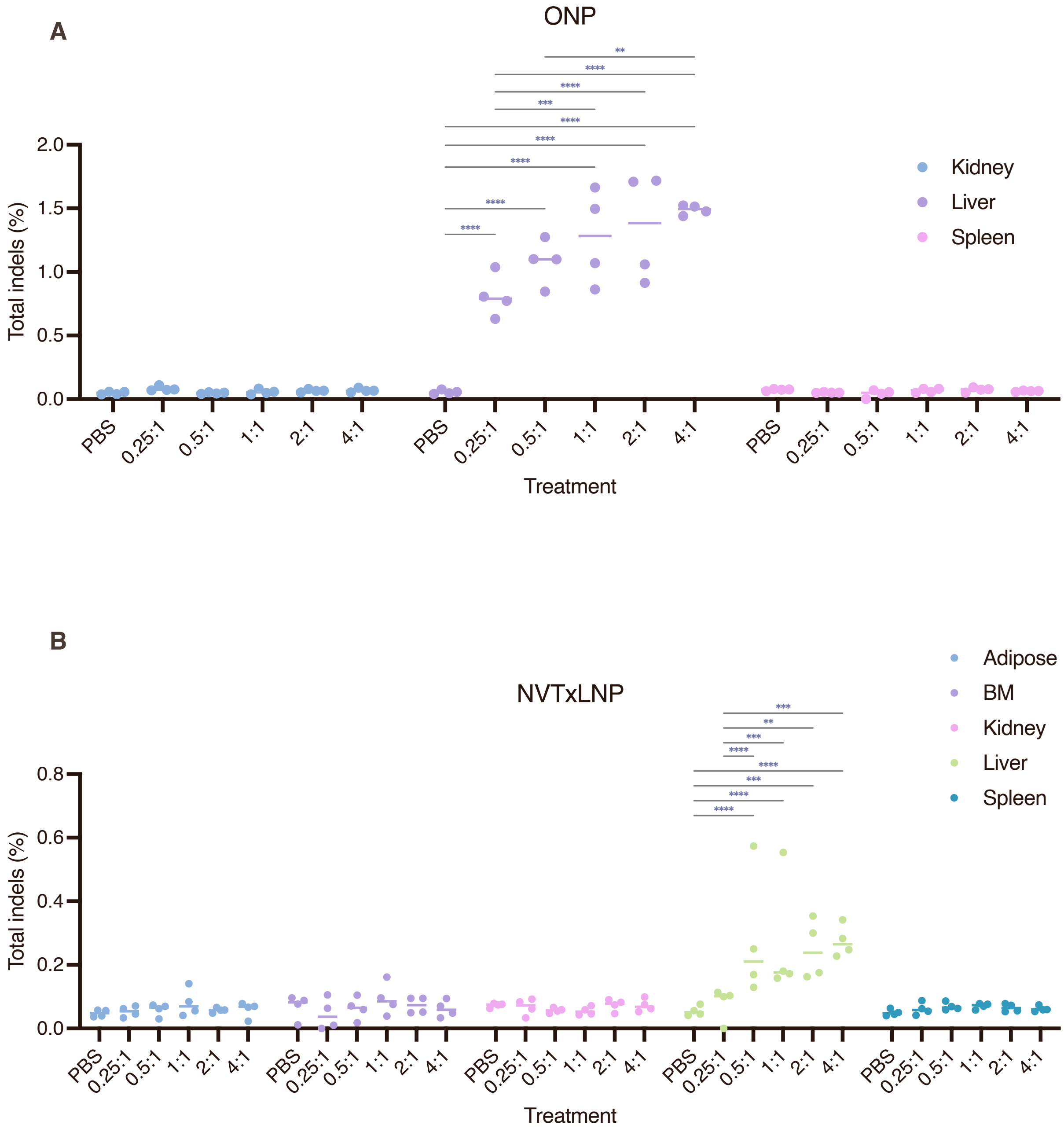

Figure S8: Percentage of reads with insertions or deletions. A) ONP-treated mice B) NVTxLNP treated mice. Indels calculated with CRISPResso2 and represents the percentage of all reads with insertions or deletions. Data represents the mean. Significance determined by two-way ANOVA with Tukey's multiple comparisons test. (*P ≤ 0.05, **P ≤ 0.01, ***P ≤ 0.001, **** P ≤ 0.0001)

Table S1: Test for linear trends: PBS mice were excluded from the analysis. An ordinary one-way ANOVA with a multiple comparison test for linear trends was used.

Treatment	Organ	Linear trend	p-value
ONP	Liver	No	>0.05
	Spleen	Yes	0.0005
	Kidney	Yes	0.0005
NVTxLNP	Liver	Yes	<0.0001
	Spleen	Yes	<0.0001
	Kidney	Yes	<0.0001
	Adipose	Yes	0.0287
	Bone marrow	Yes	0.0287

Table S2: Off-target site details: COSMID was run with a no-indel query and tolerance of up to 3 mismatches and with consideration of sites with bulges. CRISTA prediction software does not factor bulges in to its calculations and up to 3 mismatches were considered. Site 1 was identified as the most likely site for off-target editing by CRISTA and was subsequently run on COSMID to determine a predictive score. Of the top five sites identified by CRISTA, site 1 had the best COSMID score. Site 2 was identified as the 5th most likely by COSMID. It had the best CRISTA score of the top 5 sites identified by COSMID. Differences between the sgRNA and off-target sequences are shown in blue font, the PAM is bolded, and As within the potential editing window of ABE8e are underlined.

	Location	Gene	Sequence	COSMID score	CRISTA score
sgRNA			CCTCATTGATTAACGCCCAG**NGG**		
Site 1	chr2 74686824-74686846	Hoxd11 exon	CCTAATTAATTAACTCCCAG**TGG**	2.34	0.52
Site 2	chr18 36685318-36685338	E230025N22Rik exon	CCTCATTCATGAAGGCCCAG**AAG**	2.27	0.22
